# Caring approaches to young, gifted music learners' education: a PRISMA scoping review

**DOI:** 10.3389/fpsyg.2023.1167292

**Published:** 2023-07-27

**Authors:** Guadalupe López-Íñiguez, Gary E. McPherson

**Affiliations:** ^1^Sibelius Academy, University of the Arts Helsinki, Helsinki, Finland; ^2^Melbourne Conservatorium of Music, University of Melbourne, Southbank, VIC, Australia

**Keywords:** care ethics, giftedness and talent, music education, music performance, PRISMA-ScR, scoping review, socio-emotional development, special needs

## Abstract

This study reviews empirical research literature that deals with existing caring approaches to nurture and educate gifted children in music. The focus on the ethics of care stems from the need to expand notions of talent development in music from a purely behaviorist focus often associated with traumatic experiences, toward a perspective that addresses socio-emotional and cultural aspects of human development across the lifespan. We employed the Preferred Reporting Systems for Systematic Reviews and Meta-Analyses for Scoping Reviews method to review literature concerning caring approaches to the upbringing and education of children gifted for music. A total of 652 records dating from the 1930s and searched via both digital databases and manually in 41 relevant journals were retrieved from which 506 were examined using our inclusion criteria. A detailed analysis process allowed the authors to include 14 studies that were organized according to sampling location, methodologies, quality appraisal, and criteria-related topics. Eleven of the studies were qualitative with a majority of these employing semi-structured interviews for data collection, while the remaining meta strategy and quantitative studies typically employed questionnaires. Salient topics covered by the selected studies included: addressing inequalities in opportunity to access gifted programs; identifying socio-emotional needs of gifted (and twice-exceptional) students; offering a nurturing environment; focusing on intrinsic motivation; developing coping strategies for overall wellbeing; and cultivating healthy attitudes toward competitions through a spirit of peer collaboration and humility. These aspects were clustered into Francoy Gagné's Differentiated Model of Giftedness and Talent regarding natural abilities, environmental, intrapersonal, and developmental catalysts that are involved in nurturing talents in gifted children. Results suggest that the existing research on caring approaches to musically gifted children's learning and development are scarce and that current knowledge is based mostly on single one-off studies rather than systematic research, and on studies that examine a selection of aspects but not adopting a larger-scale theoretical framework. This review highlights the need for more systematic, multidisciplinary, and empirically robust studies on caring approaches to musically gifted children's learning and development, and for policy developments in educational settings where acceleration programs are offered for young, gifted music learners.

## 1. Introduction

Ever since Seashore (1919) published *The Psychology of Musical Talent* in 1919, researchers have discussed various conceptions of musical ability and how musical talent can be developed during childhood (e.g., Hallam, 2010; McPherson et al., 2022) and later stages (MacNamara et al., 2006). A large focus has been placed on the environmental catalysts that enhance (e.g., Sloboda and Howe, 1999) or impede (Persson, 2000) musical development. Literature in this area has been considerably expanded across the past 25 years. Much of this literature is focused on evidence-based discourses that advocate an expansion of the focus on describing and explaining various forms of giftedness/talent (Preckel et al., 2020), or seeks to understand more precisely the most acute forms of giftedness that distinguishes musical prodigies (i.e., McPherson, 2016; and especially Gagné and McPherson, 2016).

Another important aspect in music research has focused on how to support students with special needs (e.g., Adamek and Darrow, 2010; Kivijärvi and Poutiainen, 2019) even though within this group of studies far less attention has been given to the socio-emotional needs of or specialized education provision for gifted children as compared, for example, to children who are autistic or have a physical or mental deficit that impacts on one or more aspects of their daily lives. Although approximations to the nurturing of twice-exceptional students in music (those with both high abilities and disabilities) have been theoretically discussed (e.g., Abramo, 2015; McCord, 2017), there is a gap in how special needs courses in higher music education often do not include content regarding gifted children. Such practices are often based on anti-elitist and anti-ableist unconscious bias regarding gifted students (e.g., Brown et al., 2005; Moltzen, 2009).

In light of this, the focus of this article is on research concerned with children who are highly gifted and, specifically, how their individual needs and expectations might be best served when they experience a musical education that is focused on their social and emotional needs, as well as their access and opportunity to undertake a highly specialized training program that develops their physical and mental skills as musicians in caring manners that safeguard their wellbeing and childhood rights. Within this understanding, Gagné's (2021) developed his Differentiated Model of Giftedness and Talent (DMGT) to define the range of natural abilities, environmental, intrapersonal, and developmental catalysts that play a part in the nurturing of young, gifted music learner's talents. In this model, it is assumed that children can be considered gifted for music when they display unusually precocious “intellectual, creative and/or physical maturity well before the majority of their peers” (Gagné, 2021, p. 77). Not only is Gagné's model used by researchers internationally as a guiding framework for research on multiple dimensions of giftedness and talent, but it also forms the basis of curricula and educational policies that are used in a number of school systems worldwide (VanTassel-Baska, 2022).

## 2. Expanding approaches to the education of young, gifted music learners

For nearly a century, the general conceptual landscape on giftedness and talent in music has been one of the most controversial and puzzling topics for scholars and practitioners alike. To date, much of the literature has focused either on the acquisition of skills or on exclusively environmental factors within selected contexts to define high abilities, as well as the intra- and inter- personal factors that explain the development of high levels of musical talent. Examples include the pioneering work of John Sloboda, Michael Howe, and Jane Davidson with students in specialist music schools in England during the 1990s. Overall, their set of studies (e.g., Howe et al., 1998; Sloboda and Howe, 1999) highlighted the importance of establishing specific quantities of practice, and also the role of environmental catalysts—and especially teachers and parents—in helping to stimulate and sustain the high levels of practice needed in order to acquire high levels of musical skill. Yet, their work did not cover the types of variation in individual achievement due to personal musical aptitudes (see Gagne, 1999) or the types of educational opportunities that would cater for the specific needs of individual learners (Davidson and McPherson, 2017).

In addition, the study of the acquisition of skills and the development of musical abilities in young children (e.g., Hallam, 2010), has been an important topic for some disciplines (psychology, neuroscience), but has often taken a behaviorist position oriented toward exclusively developing cognitive-motoric characteristics, or how to recognize giftedness at increasingly early stages. This has led to maximizing gifted children's potential through acceleration programs purely oriented toward skill acquisition and domain-specific expertise (e.g., Haroutounian, 2002). At the same time, most of these studies have taken a Global North-focus and narrow emphasis on either a biographical account of a single musician, collections of interview materials from a handful of selected individuals, narrative descriptions of the traumatic damage caused to them by abusive tutors/teachers, or by exposure to society at an early age (e.g., McPherson, 2016; Salasuo et al., 2017).

Because of neoliberal practice in professional education and the age of measurement we live in (e.g., Kohn, 1992; Biesta, 2010), conceptions of musical giftedness have rarely been connected to critical discourses about disadvantaged groups of learners with special needs, or the responsibilities of music schools, music universities, and music conservatories in 21st century societies toward their healthy, all-rounded development (besides and beyond the acquisition of talent at any cost). Typically, the education of young, gifted music learners has been based on widely spread reductionist and stereotypical beliefs concerning giftedness/talent, wherein the notion of childhood ethics is missing from the notion of gifted child (e.g., Beauvais and Higham, 2016). Thus, when a child demonstrates an exceptional ability in music—e.g., cognitive, creative, affective, sensorimotor—, the socioemotional troubles tend to be seen as an inevitable side effect inherent to their persona (Gagné and McPherson, 2016). Such learners are typically conceived of as successful stars from elitist backgrounds who are capable of coping by themselves and for whom no additional support is needed (Brown et al., 2005; Moltzen, 2009).

Remarkably, and in distinct contrast to advances that have been made in defining and explaining the acquisition of musical and other forms of human accomplishment, only a few theoretical attempts have been made to envisage an inclusive education for the gifted (Slote, 2013). Apart from a few theoretical descriptions (Savage, 2006; Abramo and Natale-Abramo, 2020), little is known about the applicability of caring educational ecosystems in the nurturing of young, gifted music learners (e.g., Kenneson, 2002), and what kind of institutional changes are required to develop such ecosystems. Moreover, the contemporary reflective practitioner discourse has not fully recognized the need for such professional music education practice in which technical expert knowledge is not seen as the ultimate end but “the mediating means in the service of human good that needs to be guided by” what López-Íñiguez and Westerlund (in press) refer to, in the giftedness context, as “moral wisdom.” In line with Smith (2006), the work of these music researchers in particular has identified several gaps in the existing literature on giftedness/talent that do not attend to the ecological agendas of human development in the 21st century (UNESCO, 2019; Barnett and Jackson, 2020) and can, therefore, jeopardize the ethics of care and moral values—ultimately compromising various socio-emotional, cultural, and educational aspects of the development of children gifted for music.

Our own view is that the professional education of gifted young music learners should be seen as a special case in terms of social justice and children's rights, requiring professional care and reflexivity from these children's music teachers, caregivers, educational institutions, and nations. Accordingly, a music education is most effective when it nurtures the wellbeing of the musically precocious child through a transformative politics of care in professional music education (López-Íñiguez and Westerlund, in press) where more specialist knowledge, socioemotional support (Manturzewska, 1992), and ethical empathic approaches to the educational process is provided (Smith, 2006). Such an approach follows the legal imperatives of the United Nations Convention on the Rights of the Child (brief version in UNICEF, 2010), where it is stated that children gifted for music ought to have a future as healthy and agentic individuals.

## 3. Purpose of the study

With the above in mind, a scoping literature review was undertaken to identify any existing gaps in knowledge, as well as to systematically map potential empirical research that has been completed in this area (on an international basis) that would support caring practices in the upbringing and education of young, gifted music learners. Thus, following exploratory research questions were formulated:

1. What is known from the literature about caring education approaches that have empowered and supported gifted and talented underage music students to live as agentic and healthy individuals while pursuing desirable educational outcomes?
2. Can this knowledge be organized systematically using Gagné's (2021) Differentiated Model of Giftedness and Talent (DMGT)?
3. Is this organization helpful in identifying potential research gaps in the care ethics area in relation to gifted education in music?

This knowledge was deemed important for promoting reflexive thinking in professional musicians and institutions for gifted education in music, so that technically oriented talent education embraces ethical professionalism through “an ethics of care” (Slote, 2013).

## 4. Method

### 4.1. Description of the process

For this study, we employed the Preferred Reporting Items for Systematic Reviews and Meta-Analyses for Scoping Reviews (PRISMA-ScR; see Tricco et al., 2018) method to review publications on caring approaches to gifted education in music. This type of literature review was chosen because the topic at hand had not been previously explored and the authors were aware that the amount of research identified would not be large, and not too heterogeneous methodologically, for which a systematic review would not be possible. Scoping reviews allow researchers to identify available evidence on a given topic, analyze knowledge gaps, as well as to clarify existing concepts and their key characteristics (e.g., Tricco et al., 2018; Peters et al., 2020).

Our *a priori* protocol was drafted using the Preferred Reporting Items for Systematic Reviews and Meta-analysis Protocols (PRISMA-P; see Shamseer et al., 2015), which was revised by the research team across multiple meetings before undertaking the scoping review. The protocol attended to exclusion and inclusion criteria related to the research questions, as explained below. All stages of this scoping review, such as the final screening, data extraction, and critical appraisal were blinded and completed by two both authors independently.

We used relevant international databases in English and Spanish, with the first identified article in 1931 through to December 2022. We did not apply date boundaries for the searches in databases because there was no evidence indicating that studies concerning caring approaches to musically gifted students' education had been reported during a specific period. The search in digital databases was completed by the first author and a research assistant in December 2022 and yielded 532 records (Scopus: 81; ARSCA: 218; Eric: 132; PsycINFO: 70; Web of Science: 28; sCielo-Latindex: 3).

In addition, the first author manually checked all papers published in 41 specialist journals from the fields of music education, music psychology, and gifted education since 1990s when available. The manual searches of specialist journals took place in January 2023 and uncovered 120 records that were included for the screening (see Table 1 for details). After removing 146 duplicates, the titles, and abstracts of 506 records were screened. Of the screened records, 439 were removed as they were irrelevant (e.g., not related to music; not related to caring approaches to the education of students gifted for music; book review, academic presentation slides, or commentary; full text unavailable).

**Table 1:** Terms, search dates, databases, and specialist research journals used in the literature search in English (EN) and in Spanish (ES).

SearchTerms	music AND gifted^*^ AND talent^*^ AND care OR moral OR ethics OR “care ethics” OR empathy OR sympathy OR benevolence OR altruism OR “moral imagination” OR serving OR “ethic^*^ sensitivity” OR holistic OR growth OR “moral sensitivity” OR “interpersonal sensitivity” OR “wellbeing” OR “well-being” OR development^*^ OR “social” OR motivation^*^ OR emotion^*^ OR buoyancy OR resilience OR adaptability OR autonomy OR relatedness OR “mastery experience^*^” OR “self-efficacy” OR “self-esteem” OR “self-confidence” OR “mental health” OR “counseling” OR “counseling” OR “socializing” OR “socializing” OR preventing OR “social intelligence” OR “social interaction” OR equity OR inclusion OR rights OR “child' rights” OR “human rights” OR “safety” OR “freedom” OR “support^*^ parent^*^ style” OR “emotion^*^ support” OR enjoy^*^ OR “socioaffective development” OR nurtur^*^ OR “student centered” OR “learner centered” OR “student centered” OR “student” OR “learner centered” OR “constructivism” OR support^*^ OR teamwork OR collaboration
	Databases and Dates ofSearches	December 27^th^, 2022: • ERIC • PsycInfo • Scopus • Web of Science December 28^th^, 2022: • sCielo-Latindex January 16^th^, 2023: • ARSCA–Uniarts Helsinki Central Discovery Index database, which accessed following sources: ♢ Alexander Street Press (Music Online); ♢ CCC Get It Now (Alexander Street Press; Sage Journals Online; Springer; Taylor and Francis; Wiley Blackwell; Wolters Kluwer); ♢ DOAJ; ♢ EBSCOhost (Academic Search Elite; International Bibliography of Theater and Dance with Full Text; Free E-journals); ♢ JSTOR; ♢ Oxford University Press (Oxford Handbooks Online Music; Oxford Music Online); ♢ Project MUSE Premium Collection; ♢ ProQuest (Art, Design and Architecture Collection; Dissertation and Theses; Central; Music Periodicals Database; Performing Arts Periodicals Database; Publicly Available Content Database); ♢ PubMeb (Central; Open Access); ♢ SAGE Complete; and ♢ Taylor and Francis eBooks Complete
		Specialist Research Journals and Dates of Searches^*^	January 3^rd^, 2023: 1. Action, Criticism, and Theory for Music Education (EN) hspace 2. Australian Journal of Music Education (EN) hspace 3. British Journal of Music Education (EN) hspace 4. Bulletin of the Council for Research in Music Education (EN) hspace 5. International Journal of Music Education (EN) hspace 6. Journal of Music Teacher Education (EN) hspace 7. Journal of Research in Music Education (EN) hspace 8. Musicae Scientiae (EN) hspace 9. Music and Science (EN) January 4^th^, 2023: 10. Frontiers in Psychology–Performance Science (EN) hspace 11. Music Educators Journal (EN) hspace 12. Music Education Research (EN) hspace 13. Music Performance Research (EN) hspace 14. Psychology of Music (EN) hspace 15. Research Studies in Music Education (EN) hspace 16. Visions of Research in Music Education (EN) January 7^th^, 2023: 17. British Journal of Ethnomusicology (EN) hspace 18. Epistemus. Revista Científica sobre Estudios en Música, Cognición y Cultura (ES) hspace 19. Ethnomusicology Journal (EN) hspace 20. Faisca: Revista de Altas Capacidades (ES) hspace 21. Finnish Journal of Music Education (EN) hspace 22. Nordic Research in Music Education (EN) hspace 23. Revista Complutense de Educación Musical (ES) hspace 24. Revista Electrónica de Educación Musical (ES) hspace 25. Revista Internacional de Educación Musical (ES) hspace 26. Revista Latinoamericana de Educación Inclusiva (ES) hspace 27. The Journal of Musicology (EN) hspace 28. Update: Applications of Research in Music Education (EN) January 9^th^, 2023: 29. European Journal of High Ability (EN) hspace 30. Exceptional Children (EN) hspace 31. Gifted Child Quarterly (EN) hspace 32. Gifted Child Today (EN) hspace 33. Gifted Education International (EN) hspace 34. High Ability Studies (EN) hspace 35. Journal for the Education of the Gifted (EN) hspace 36. Journal of Gifted Education and Creativity (EN) January 12^th^, 2023: 37. Australasian Journal of Gifted Education (EN) hspace 38. Gifted and Talented International (EN) hspace 39. Journal of Advanced Academics (EN) hspace 40. Learning and Individual Differences (EN) hspace 41. Roeper Review (EN)

^*^Manual searches since 1990s when available.

Both authors retrieved the full texts of the remaining 67 records and independently examined these for their eligibility based on the following inclusion criteria: (1) full text was available and written in English or Spanish; (2) the publications included original research articles, data-driven book chapters, or doctoral dissertations; and (3) caring approaches to gifted education in connection to music was either the topic, a central element of the study, or a discussion point derived from the findings/results. Details of how the searches were conducted are shown in Table 1.

A total of 53 full-text studies were excluded due to the following reasons: (1) non-empirical studies (purely theoretical); (2) unclear or inadequate design, sampling, or document type; (3) exclusive focus on talent development; (4) utilizing music for the overall learning and development of academically gifted students in general education settings; (5) exclusive focus on identification, admission, and provision of acceleration programs; (6) describing environmental influences on technically-oriented talent development; (7) defining gifted people's characteristics; and (8) exclusive focus on describing trauma without offering solutions. Findings of the 14 studies included in the final analysis are described in the Findings section.

### 4.2. Analytical approach

**Figure 1:**
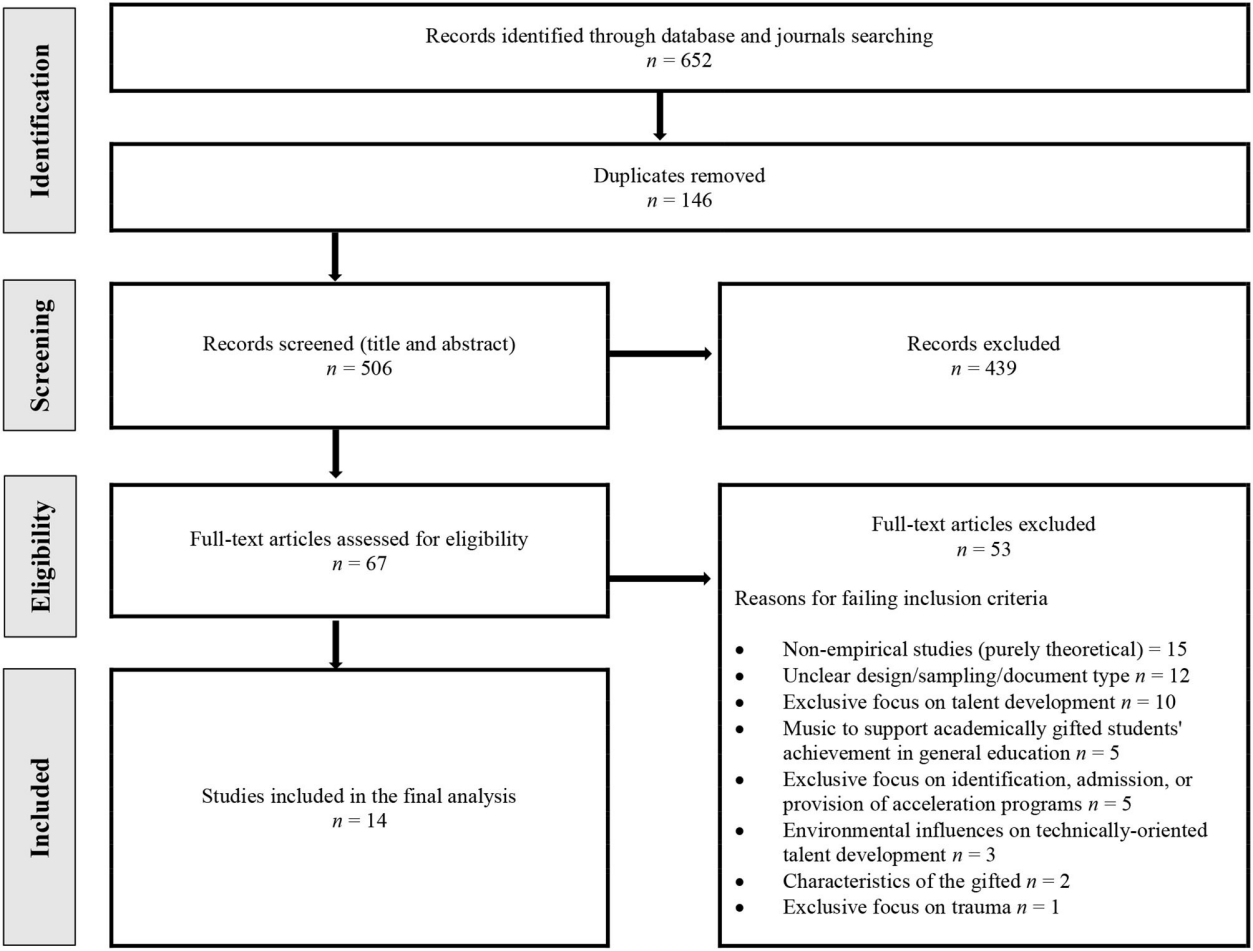
Process of selecting studies following the PRISMA flowchart (Moher et al., 2009).

The research topic was defined based on existing gaps in a preliminary literature search. This was followed by the selection of relevant studies using the PRISMA flowchart (see Figure 1). Within this phase, we undertook a scoping review of relevant literature and drew up eligibility criteria for selecting the studies to be reviewed. For this, we used English and Spanish variations of the search terms *music*, gifted^*^ and *talent* in combination with relevant keywords (see Table 1 for details).

The 67 results of the search that were eventually included in the first screening stage concerned caring aspects within music education. With the screening process in Covidence, the second author blindly checked 100% of the full-text articles assessed for eligibility, and 6 inconsistencies against the selection criteria for excluded papers were found, which helped refine the criteria. After this, we read the studies to be included in the review and extracted the data by appraising the quality of their research questions and methods using the Mixed Methods Appraisal Tool (Hong et al., 2018). We also conducted a thematic content analysis to identify codes across the 14 studies that are presented in the next section.

## 5. Findings

### 5.1. Overall publication trends

The 14 studies included in this review were conducted in Singapore (*n* = 3), the United States (*n* = 2), and in Australia, Estonia, France, Germany, Finland, Norway, Poland, Romania, Taiwan, and the United Kingdom (*n* = 1). A total of 1,944 participants took part in the 14 studies, of whom over 1,200 were music students (estimate, as the sampling information in some studies was insufficient to make an exact calculation). The remaining participants included music teachers, ceased or alive professional performing musicians, parents, band directors, or students of other arts or academic disciplines.

Most of the studies were qualitative (*n* = 11), with the remainder either quantitative (*n* = 3), or mixed methods (*n* = 1). Data were collected via surveys (2 studies), semi-structure and standardized questionnaires (*n* = 7), interviews (8 studies), case studies of diverse nature (11 studies), observations (3 studies), designs with control groups (2 studies), essays (1 study), and other means such as phone conversations and email correspondence, review of documents and blog entries, or photographs (*n* = 2).

The selected studies were published in journals related to gifted education (*n* = 7), music education (*n* = 3), music psychology (*n* = 2), and the learning sciences (*n* = 2) between the years 2000 and 2020. Using the quality appraisal categories developed by Dixon-Woods et al. (2007), we did not find any paper deemed flawed or irrelevant. However, one (1) of the articles was appraised as a key paper, seven (7) of the studies were assessed as being of satisfactory quality, and the remaining six (6) papers' relevance was unclear. All the information can be seen in Table 2.

**Table 2:** Reading the studies to be included in the review and extracting the data.

**No**.	**Author(s) (year), journal**	**Title of study**	**Design**	**Country**	**Participants**	**Sample**	**Data collection**	**Quality appraisal**	**Caring approach**
1	Coppola (2019) *Bulletin of the Council for Research in Music Education*	Musical humility: An ethnographic case study of a competitive high school jazz band	Qualitative	United States	High school students (11) and band director (1)	12	Ethnographic case study, semi-structured interviews, observations	Satisfactory	Humility Collaboration, and egalitarian practices
2	Freeman (2000) *Roeper Review*	Children's talent in fine art and music – England	Mixed methods	United Kingdom	Primary school students (12 from music)	72	Experimental design (including 2 control groups), questionnaires	Satisfactory	*Gifted Education Provision for Disdvantaged Children*
3	Garces-Bascal et al. (2011) *Gifted Child Quarterly*	Soul behind the skill, heart behind the technique: Experiences of flow among artistically talented students in Singapore	Qualitative	Singapore	Specialized-arts secondary school students (3 from music)	14	Case studies, interviews, semi-structured questionnaire	Unclear relevance	*Artistic Sensibility Personal Connectedness*
4	Garces-Bascal (2013) *Roeper Review*	Perceived family influences in talent development among artistically talented teenagers in Singapore	Qualitative	Singapore	Specialized-arts secondary school students (3 from music)	14	Case studies, interviews, semi-structured questionnaire	Unclear relevance	*Childrearing (*parental styles and practices)
5	Gembris et al. (2020) *Frontiers in Psychology–Performance Science*	High-performing young musicians' playing-related pain. Results of a large-scale survey	Quantitative	Germany	Underage and adult musicians participating at the national level of the “Jugend musiziert” contest	1.143	Exploratory design, survey including open-ended questions, standardized questionnaires	Satisfactory	Coping and wellbeing (health and pain prevention)
6	Haraldsen et al. (2020) *Roeper Review*	Thriving, striving, or just surviving? TD learning conditions, motivational processes and well-being among Norwegian elite performers in music, ballet, and sport	Qualitative	Norway	Former students at the Norwegian talent development schools (pre-college level; 3 from music)	9	Narrative design, lifespan perspective, semi-structured interviews	Unclear relevance	Self-determined motivation (focus on intrinsic motivation)
7	Hendricks and McPherson (2010) *International Journal of Music Education*	Early stages of musical development: Relationships between sensory integration dysfunction, parental influence, and musical disposition of a 3-year-old “maestro”	Qualitative	United States and Australia	Preschool child (1) and parents (2)	3	Case study, interviews, observations, questionnaires, journal, and blog entries, photographs, email correspondence	Satisfactory	*Childrearing (*parental styles and practices) *Twice Exceptionality*
8	Ho and Chong (2010) *International Journal of Music Education*	The talent development of a musically gifted adolescent in Singapore	Qualitative	Singapore	Gifted education programme and music conservatory student (1), parents (2), and music teachers (2)	5	Case study, lifespan perspective, survey, and interviews	Key paper	*Childrearing (*parental styles and practices) *Nurturing teachers*
9	Jung (2015) *The Australasian Journal of Gifted Education*	Unfulfilled potential: The adult careers of former musical prodigies Ervin Nyiregyházi, Fanny Mendelssohn Hensel, and David Helfgott	Qualitative	Australia	Ceased musical prodigies	3	Biographical case studies	Unclear relevance	Holistic approach to nurturing children gifted for music
10	Kao (2011) *High Ability Studies*	The dilemma of competition encountered by musically gifted Asian male students: An exploration from the perspective of gifted education	Qualitative	Taiwan	Junior high school students participating in gifted music programs (7), their mothers (7) and music teachers (7)	21	Multiple-case study, semi-structured interviews, observations, phone conversations, and review of documents	Satisfactory	*Cultivating healthy attitudes toward competitions (psychosocial coaching)*
11	Nogaj (2017) *Polish Psychological Bulletin*	Locus of control and styles of coping with stress in students educated at Polish music and visual arts schools–a cross-sectional study	Quantitative	Poland	Students from schools with professional arts programs (*n* for music unknown)	354	Cross-sectional study (including 1 control group), standardized questionnaires	Unclear relevance	*Coping and wellbeing*
12	Rucsanda et al. (2020) *Psychology of Music*	Musical performance and emotions in children: The case of musical competitions	Quantitative	Romania	Children and teenagers participating in international music competitions for young musicians	146	Correlational study, standarized questionnaire	Satisfactory	Cultivating healthy attitudes toward competitions (psychosocial coaching) *Psychological/emotional training*
13	Ruokonen et al. (2011) *Procedia-Social and Behavioral Sciences*	“They have always supported my choices.” Creative catalysts in university students' learning environments	Qualitative	Finland and Estonia	Estonian and Finnish university students especially talented in music or other arts	115	Comparative, phenomenographic multiple-case study, lifespan essays	Unclear relevance	*Childrearing* (parental styles and practices) Self-determined motivation (focus on intrinsic motivation) *Nurturing teachers*
14	Tordjman et al. (2020) *Journal for the Education of the Gifted*	Rethinking human potential in terms of strength and fragility: A case study of Michael Jackson	Qualitative	France	Ceased musical prodigy	1	Biographical case study	Unclear relevance	*Socioemotional needs and development*

### 5.2. Grouping of the selected studies and identification of topics

Each of the 14 articles covered different facets and often multiple dimensions that are directly or indirectly associated with our central concern of caring for young, gifted music learners. These included: humility, collaboration and egalitarian practices, gifted education provision for disadvantaged children, artistic sensibility, personal connectedness, childrearing (parental styles and practices), coping and wellbeing (health and pain prevention), self-determined motivation (focus on intrinsic motivation), twice exceptionality, nurturing teachers, holistic approach to nurturing children gifted for music, cultivating healthy attitudes toward competitions (psychosocial coaching), psychological/emotional training, and socioemotional needs and development.

These 14 identified caring approaches have a higher level of abstraction and represent the themes that were derived from the coding process. The initial level of coding in our narrative analysis included broader codes such as childrearing values, supportive and nurturing parents, developmental care and readiness, learner-centeredness and intrinsic motivation, or brief discussion on a holistic approach to nurturing children gifted for music. These led to the 14 themes enumerated above in a second analysis stage. Table 3 shows how these themes clustered into the natural abilities and environmental, intrapersonal, and developmental catalysts depicted in Gagné's (2021) Differentiated Model of Giftedness and Talent (DMGT).

**Table 3:** Comparison between the aspects of development comprised in Gagné's (2021) DMGT model and the identified topics found in the selected studies.

**Gagné's model**	**Main dimensions of the model**	**Articles that relate to these dimensions of development**
**Natural abilities**	Mental–intellectual, creative, social, perceptual Physical–muscular, motor control	Twice exceptionality (Hendricks and McPherson, 2010) Artistic sensibility (Garces-Bascal et al., 2011)
**Environmental catalysts**	Milieu–social, familial, environment (support mechanisms to reinforce familiarity, comfort, satisfaction with living and musical environment) Individuals and others–influence of parents, family, peers, teachers, mentors Provisions–enrichment programs provided, curriculum, form of pedagogy, pacing/pushing, acceleration, catering for needs	Gifted education provision for disadvantaged gifted children (Freeman, 2000) Childrearing (parental styles and practices) (Hendricks and McPherson, 2010; Ho and Chong, 2010; Ruokonen et al., 2011; Garces-Bascal, 2013) Nurturing teachers (Ho and Chong, 2010; Ruokonen et al., 2011) Holistic approach to nurturing children gifted for music (Jung, 2015) Collaboration and egalitarian practices (Coppola, 2019) Socioemotional needs and development (Tordjman et al., 2020)
**Intrapersonal catalysts**	Physical–appearance, handicaps, health Mental–temperament, personality, resilience Awareness–self and others, strengths, and weaknesses Motivation–values, needs, interests, passions Volition–autonomy, effort, perseverance, buoyancy, adaptability	Coping and wellbeing (health and pain prevention) (Nogaj, 2017; Gembris et al., 2020) Self-determined motivation (focus on intrinsic motivation) (Ruokonen et al., 2011; Haraldsen et al., 2020) Humility (Coppola, 2019) Personal connectedness (Garces-Bascal et al., 2011) Psychological/emotional training (Rucsanda et al., 2020)
**Developmental process**	Activities, including access, content, formal/informal Investment, including time, energy, money Progress, including stages, pacing, and turning points (special events that provide mastery experiences)	Cultivating healthy attitudes toward competitions (psychosocial coaching) (Kao, 2011; Rucsanda et al., 2020)

### 5.3. Synthesis of the studies

Following PRISMA-ScR guidelines, the selected studies of this research did not allow for quantitative synthesis. Thus, an outline of the main emphasis and findings of each of these studies is presented briefly in the following sub-sections. The studies are presented in alphabetical order.

#### 5.3.1. Study 1: “musical humility: an ethnographic case study of a competitive high school jazz band”

The case study presented by Coppola (2019) examines the role of humility within a competitive high school jazz band in the United States. Musical humility was theorized to encompass: “(a) purposeful musical engagement and collaboration, (b) lack of superiority, (c) acknowledgment of shortcomings and learnability, (d) other-orientedness, and (e) healthy pride” (p. 9). Two implications arising from the research suggest that teachers might embrace this framework for fulfilling two sociomusical imperatives in their teaching. First, as a means of practicing humility within their musical interactions more broadly, and second as a by-product of egalitarian participation in and through music more generally. For the author, “this nascent construct could come to simultaneously illustrate how humility might improve musical experiences and how musical participation might be harnessed to promote humility in turn” (p. 22).

#### 5.3.2. Study 2: “children's talent in fine art and music–England”

The study by gifted education scholar Joan Freeman (2000) examines outstanding talent in children in music and fine art. It incorporates measures to provide evidence that aesthetic perceptual development begins at birth, then becomes habitual, and subsequently shapes future interest and participation. Home environment was particularly important even when the child is exposed to high level provisions at school. In addition, Freeman concluded that some of the schools failed to produce any children of assessable talent and that huge discrepancies existed between the provisions provided for artistic development across schools. This led her to express difficulty in accepting that instances of exceptional talent are definable by school catchment. Importantly, this research stresses the need for future research to focus on contexts, most important of which are the wider social implications of how communities value the arts more generally and understand through their actions the important role of the arts for transmitting cultural values, developing a child's perceptual and analytical abilities, and nurturing the types of creativity and imagination required for innovative thinking and high-level problem solving.

#### 5.3.3. Study 3: “soul behind the skill, heart behind the technique: experiences of flow among artistically talented students in Singapore”

Garces-Bascal et al. (2011) present case studies of 14 Singaporean students enrolled in a specialized secondary school who were asked about their experiences of flow as they undertook training in visual arts, dance, music, and theater. Results reveal that the learners experienced certain forms of flow through establishing clear goals for themselves, experiencing intense concentration, enjoyment and loss of self-consciousness, and transformation of time. Implications for teaching were grouped around five themes:

“(1) the significance of investing a sense of personal connectedness with the students as they engage in their training, (2) the importance of immediate feedback as they progress in their respective fields, (3) the inspiration that is derived from seeing the students' own mentors perform or, at the very least, having a firsthand knowledge of what it means to be in that domain or the transfer of tacit knowledge and insider information […], (4) the sense of kinship that is fostered in a school that allows one a measure of safety to express one's artistic sensibilities, and (5) the balance between “fun” and rigor that engages and captures the students' attention” (p. 205).

#### 5.3.4. Study 4: “perceived family influences in talent development among artistically talented teenagers in Singapore”

Using the same participants and in the previous study, Garces-Bascal (2013) examines how Singaporean families promote literacy and instill values in academic excellence within the home environment. Results show how families influence the talent development process and their involvement in their child's interest and involvement in the arts activity in which they are specializing. Parents' style and practices are shaped by their occupational backgrounds and reflect patterns of childrearing that are common within Asian contexts.

#### 5.3.5. Study 5: “high-performing young musicians' playing-related pain. Results of a large-scale survey”

The recent publication by Gembris et al. (2020) presents a large sample study that deals with how playing-related pain (PRP) impacts on the development of highly talented young musicians by addressing how they cope, behave, and communicate about PRP with other young musicians, their teachers, their parents, and others, including their peers. Results show that around three quarters of the sample reported that PRP impacted on their development and that females were significantly more affected than their male counterparts. Importantly, duration of practice and prevalence of PRP were closely associated. Just over half of the young musicians reported being taken seriously about their PRP, but alarmingly around 44% felt that their complaints were not taken seriously enough or ignored. The study confirms that around three quarters *(75%*) of highly talented young music learners will be typically impacted by PRP and recommends ways of counteracting this problem through teaching more adequate practicing techniques that might involve, for example, warm-up and cool-down activities, breaks, correcting incorrect posture, and self-observation while practicing. The researchers cite alarming evidence showing that some parents believe that PRP is an inevitable problem that talented young musician must endure. The authors therefore assert that these types of opinions need to be confronted and should not be accepted. One way of achieving this is for the teacher to include information about health and prevention in all lessons. For these researchers, better communication between learner, teacher, and parents is fundamental to avoiding PRP in talented young musicians.

#### 5.3.6. Study 6: “thriving, striving, or just surviving? TD learning conditions, motivational processes and well-being among Norwegian elite performers in music, ballet, and sport”

Haraldsen et al. (2020) interview nine elite performers in ballet, music, and swimming to examine their motivational orientation at Norwegian talent development (TD) schools to understand how the performers' development was shaped by egalitarian values, high-performance deliberate practice, and controlling conditions. Results reveal multifaceted motivational profiles, ranging from predominantly self-determined to predominantly be controlled. Performers regulated by self-determined motivation experienced more joyful, robust, and healthy development that was characterized by self-realization, flow, self-esteem, and vitality. They were also less dependence on their TD learning conditions. Performers who experienced a more controlling learning environment reported higher vulnerability, and ill-being that was evident in low self-esteem, perfectionism tendencies, obsessiveness, stress, negative affect, and exhaustion. In contrast to other literature this study reports that many of the performers did not possess an original intrinsic motivation with only two of the nine performers displaying a predominantly autonomous engagement. Based on the findings, the authors assert that some of the negative aspects of the performers' TD include over-striving to compensate for low levels of self-worth, and perfectionism in the form of striving to become an “ideal-self.” They also pointedly remark that “In a debilitative circle of negative emotion (frustration, negative affect, and stress), cognition (guild, shame, and performance anxiety), and behavior (rigidity, obsession, and eating disorders), the performers' self seemingly will become diminished” (p. 121). To alleviate these tendencies the authors warn that TD programs must guard against the negative consequences of controlling environments, and strive to reinforce and enhance the performers' autonomous motivation and their self-determined psychological needs of relatedness, competence, and especially autonomy.

#### 5.3.7. Study 7: “early stages of musical development: relationships between sensory integration dysfunction, parental influence, and musical disposition of a 3-year-old ‘maestro”'

The article by Hendricks and McPherson (2010) presents a case study of a 3-year-old boy with neurological disorder (Sensory Integration Dysfunction) and the parent-child interactions that facilitated his involvement in music from an early age. Stressed is the high level of attention and support the child is provided with from his parents, in terms of an elongated period of “communicative musicality” that is characteristic of mother-infant bonding processes during infancy. Specifically, interactions involving both child and parents resulted in all participants developing competence, connectedness, and their sense of musical identity through “organic synchronous and reciprocal interactions […] in freely creative settings” (Custodero, 2009; p. 514). The study concludes that important work is yet to be undertaken on how these interactions might help with our understandings of the acquisition of precocious musical involvement during infancy and early childhood.

#### 5.3.8. Study 8: “the talent development of a musically gifted adolescent in Singapore”

The study by Ho and Chong (2010)—which was the only one assessed as a key paper for this literature review—applies Gagné's model to examine aspects of natural ability and environmental catalysts that related to talent development within the Singaporean context. Citing Sternberg (2007), the authors suggest that “giftedness represents a set of cultural values” (p. 57) and that differing values, customs, and beliefs related to notions of giftedness need to be recognized. In this study, the home environment of the gifted learners studied typically offered opportunities for exposure to multiple rigorous and challenging forms of musical engagement at each age that greatly enhanced their musical development. Consequently, one of the significant findings that emerged is the wealth of cultural knowledge and resources that are available in the homes of the musically gifted young Singaporean musician through their interactions with others, particularly their parents. The authors show how musicians can access cultural knowledge and values of a type that are invaluable for improving young musicians' motivation to participate in and develop their musical potential. Stressed is the need for music educators to consider home-school relationships in their quest for fostering links between their own teaching strategies and the lived experiences of their students.

#### 5.3.9. Study 9: “unfulfilled potential: the adult careers of former musical prodigies Ervin Nyiregyházi, Fanny Mendelssohn Hensel, and David Helfgott”

The study of Jung (2015) draws on the lives of three notable musical prodigies (Ervin Nyiregyházi, Fanny Mendelssohn Hensel, David Helfgott) who sought out musical careers but did not experience adult success despite their extraordinary musical potential. Interestingly, none of the reason their adult careers floundered relate to their musical abilities but rather a less than optimal level of psychosocial skill (Nyiregyházi), a restrictive societal and family environment (Hensel), and mental illness (Helfgott). This study is important because it highlights how exceptional natural musical abilities interact with and compliment various non-musical attributes. Examples would include access to high quality music teachers, consistent family support, participation in a community or culture that supports the transition from musical potential to talent, and the possession of personal qualities related to perseverance and discipline, in addition to sound interpersonal skills. Also important are access and opportunities to take advantage of chances that will facilitate a career, freedom from issues (e.g., health) that might impact on future commitment to succeed, in addition to a multitude of other non-musical factors that have not yet been identified and described because of a limited research base.

#### 5.3.10. Study 10: “the dilemma of competition encountered by musically gifted Asian male students: an exploration from the perspective of gifted education”

Kao (2011) examines competitiveness in musically gifted students across the daily experiences of seven musically gifted Taiwanese adolescent males who were participating in a competitive music class. Five themes emerged from the study: (1) tacit student competition and rivalry; (2) a tendency for students not to discuss music between themselves; (3) utilitarian training where the music students spent most of their time practicing very few pieces of difficult repertoire to acquire a competitive edge for “big occasions”; (4) for some students, feelings of inadequacy due to lower levels of mental maturity and self-discipline, or denial of natural giftedness by other classmates; and (5) and critical comparisons made by parents and teachers who pushed their child or students to achieve at a higher level through ongoing comparisons with others. The author concludes that gifted students would benefit from psychosocial coaching to activate their intrinsic motivation.

#### 5.3.11. Study 11: “locus of control and styles of coping with stress in students educated at polish music and visual arts schools–a cross-sectional study”

Using a sample of music schools in Poland and Serbia the study by Nogaj (2017) provides an overview of stages of development related to musically talented children and youth. Despite cultural differences between the two countries, both share similar models for educating students in their music schools. The authors suggest that during preschool, informal and spontaneous activities should form the basis of preparing the young children for participation in more specialist instrumental/vocal training later, and that talent identification mainly occurs within the family. A “romance or from abilities to competence” (p. 156; see also Subotnik and Jarvin, 2005) which occurs when children enter Elementary school offers opportunities to learn how to play an instrument and gain tuition. Children's progression will not only depend on their individual abilities but also the forms of parental and teacher support or pressure they received in the form of external rewards and recognition. When these young learners enter high school, they move to a period in which they are developmental ready to aim for precision and perfection in their mastery of an instrument. It is also a period in which social support from parents and peers is particularly important so that the adolescent musician can cope with the pressures of learning at a high level. For those who choose higher music education, coping with pressure is dependent on integration of the musical, personal, social, and environmental influences experienced during earlier periods.

#### 5.3.12. Study 12: “musical performance and emotions in children: the case of musical competitions”

The recent study of Rucsanda et al. (2020) investigates 146 young singers who participate in music competitions to compare the emotions of those who obtained prizes with those who did not. The researchers found that lower performance quality was related to negative emotions while higher performance quality was linked to positive emotions, low arousal, and increased dominance. More experienced singers demonstrated more positive emotions, lower arousal, and high dominance. Consequently, experience in music competitions was theorized to mediate emotions and the quality of music performance in competitions. The researchers draw on evidence showing that young people become more self-critical, experience increasing levels of performance-related emotions and are increasingly capable of evaluating themselves compared to others as they progress through childhood to adolescence. Psychological and emotional training is therefore recommended to support the wellbeing of young singers so that they are more capable of understanding, expressing, and managing their emotions when preparing for and participating in music competitions.

#### 5.3.13. Study 13: “'they have always supported my choices.' Creative catalysts in university students' learning environments”

The study by Ruokonen et al. (2011) describes important environment catalysts of Estonian and Finnish university students that have contributed to their artistic giftedness and creative products or presentations. Ruokonen et al. stress the importance of home and school environments, cultural communities, and informal learning opportunities, and draw the conclusion that to aid in early development, children should be exposed to both formal and informal aspects and various environmental catalysts that connect to their intrinsic motivation and the development of their creativity and giftedness.

#### 5.3.14. Study 14: “rethinking human potential in terms of strength and fragility: a case study of Michael Jackson”

The final selected study by Tordjman et al. (2020) presents a case study of Michael Jackson's high potential, talent, and precocity that stresses the usefulness of a multidimensional approach to examining human potential. Highlighted are the asynchronies evident in extreme talent and the impaired socio-affective development and interplay between strength and fragility in a case where cognitive functioning is not able to be separated from emotional functioning. The authors show—using the case of Jackson—how exceptional qualities as a musician can be accompanied by extreme fragility that can “freeze an individual in a youthful state that may result in serious developmental maladjustments” (p. 74). Publicly recognized and admired precocity, therefore, can lead to a host of identity problems, immaturity, and socioemotional difficulties. These might result in a highly gifted child and then adolescent displaying symptoms of depression, together with a fading precocity and decrease in precociousness—which the authors believe is the result of a freeze in emotional development. Consequently, schools, society, and individual families need to ors into considerations so that children are not defined exclusively through their gift for music or other high-level form of achievement.

## 6. Discussion

### 6.1. Summary of evidence

The primary purpose of this scoping PRISMA literature review was to identify international research that reflects existing caring education approaches that can be used to empower and support gifted and talented underage music learners to live as agentic and healthy individuals while pursuing desirable educational outcomes. Our analysis of the literature reveals that there is only a superficial coverage of research on this topic, with only 14 research articles identified in our search. Close examination of the content of these 14 studies shows that they are only loosely related to aspects of “care” and minimally aligned with the natural abilities, environmental and intrapersonal catalysts, and developmental processes described within the holistic DMGT model proposed by Gagné (2021). The most important finding of this review, therefore, is a serious lack of systematic research on caring approaches to educating young, gifted musicians.

The 14 identified studies largely consist of single studies on the topic or multiple write ups using the same sample of participants as in the case of Garces-Bascal (2013) and her colleagues (Garces-Bascal et al., 2011) that cover differing aspects related to the development of 14 gifted Singaporean youth. Among the most impressive work is that by Freeman (2000) who studied highly talented English students for much of her professional life. Her studies have expanded views across the past 30 years on work undertaken in specialist English schools and the influences within the home environment that facilitate talent development. Other rigorous research has been undertaken recently by Gembris et al. (2020) on pain related problems with young musicians, as well as studies led by Rucsanda et al. (2020) and Haraldsen et al. (2020) who focus on motivational aspects related to extrinsic motivation—such as those experienced in competitions or high stakes “hot bed” learning environments. In addition, Jung (2015) details the lives of three notable prodigies who experienced a less than optimal level of psychosocial skill, a restrictive societal and family environment, or mental illness.

As is evident from the information outlined above, the existing literature provides only a superficial coverage of the multiple facets that relate to caring approaches to developing gifted young music learners' education or personal development—with no single study or set of studies specifically focused on the aspect of “caring environments” for young, gifted music learners. In this context, engaging with a caring approach would require “empathetic behaviors in both the public and domestic spaces surrounding [musically gifted] children,” and confronting “the still common patriarchal and elitist ethos of the education of [these children], while seriously considering alternative, mutually negotiated educational paths for serving the[ir] wellbeing” (López-Íñiguez and Westerlund, in press).

Of equal concern is that few of the studies identified employed a strong theoretical framework for examining issues examined in their studies. From the selected studies, the exception was the study by Ho and Chong (2010) which—using Gagné's model in their analysis, and being the only article appraised as a key paper for the purposes of our scoping literature review—tackled the ways a musically gifted child could be nurtured and socialized to thrive in the music world (Ho and Chong, 2010). In that line, an added ecological dimension to our study includes extending theory to include much broader human perspectives such as care ethics, justice, and democracy for the gifted—so that a more inclusive education for the gifted, and less imposed futures can be envisaged (e.g., Slote, 2013). This, in turn, calls for educational reforms that recognize childhood rights, by allowing gifted children to become agentic in their decisions regarding their minds and bodies, as well as empowered to realize their potential to become social agents (e.g., Wyness et al., 2004).

Although two studies dealt specifically with how music students could better navigate the competitive performing world (Kao, 2011; Coppola, 2019), a related, relevant point concerns how a young gifted child copes with success. Because the bar for outstanding performances is continuously being raised, young, gifted music students (and those who are gifted in other pursuits) need to be adaptable to cope with the “seemingly limitless extent of human performance possibilities” (Subotnik et al., 2011; pp. 12). To facilitate this adaptability, young, gifted music learner's education needs to be flexible and to feature the types of socio-emotional development components that go well beyond traditional conceptions of talent development that rely (often exclusively) on repetitive practice of a small repertoire of music literature. In this line, teachers and parents need to be more mindful about systems of rewards/punishments (see Kohn, 1992), about understanding giftedness as a synonym for “success,” and about realizing the role of chance and the environment for supporting or alternatively preventing individuals from reaching their potential in healthy or unhealthy ways (see Gagné, 2021).

In line with the above comments, we were pleased to find the Garces-Bascal et al. (2011) study that deals with artistic sensibility. However, apart from this study there is a complete lack of other research dealing with the critical pedagogical approach of linking high level performance skills with the types of creativity and innovation needed for a young, gifted music learner to perform in an original and uniquely artistic manner that goes beyond the reproductive and technical skills mentioned above. In our view, creativity and innovation are fundamental to higher levels of music performance but as is evident in this review, have received virtually no research attention regarding the development of young, gifted music learners. Unfortunately, the conservatoire tradition—which often unduly emphasizes technique and rigidity in learning—does not always lead to autonomous and artistically agentic learners (e.g., Pozo et al., 2022) nor does it promote reflexive thinking in ways that can embrace ethical professionalism through an ethics of care (Slote, 2013).

Regarding the various studies on nurturing parents/teachers, it also became clear that there are key agents in the development of these children, but no study addressed the issue of caring for young, gifted music learners who might be placed in dysfunctional environments, or who might have experienced trauma and abuse. In this case, further research would be needed to identify and better support such students to safeguard their rights. In addition, parents and teachers contribute to eminence achievement, yet encouragement and stimulation are not always necessarily accompanied by emotional support. To address this deficiency, we recommend that music education institutions should provide training for both practitioners and for families.

Although gifted young learners might be exceptional at playing music, there is a need to better assess the extent to which they engage in music due to their intrinsic motivation for making music (i.e., their passion for music making), or because of various extrinsic motivations related to external reinforcement of a type that supports (but may also push or coerce) them into maintaining their involvement in music at a high level. The delicate balance between intrinsic motivation and external forms of coercion and control needs much more research attention in the population of young, highly gifted music learners, particularly given the many reports of prodigies and gifted individuals who acknowledge being harmed by the pressures of competitions, demanding parents, uncompromising teachers, or a constant feeling of needing to excel at a level that is higher than any of their peers.

Two studies tackled the provision of gifted education for disadvantaged children, as well as caring for those students who fall under the twice-exceptionality spectrum (Freeman, 2000; Hendricks and McPherson, 2010, respectively). However, we need more nuanced studies that address inequalities in opportunity to study in gifted music programs especially in relation to minorities, underage women, and students coming from less affluent families or who live in rural contexts with less access to specialized music education for the gifted.

Finally, several key concepts related to caring are being studied in educational psychology that deserve to be applied in research on young, gifted music learners. Among the most important is work by self-determination theorists (e.g., Evans and Ryan, 2022) dealing with conditions that can undermine enjoyment and be detrimental to motivation and wellbeing, particularly when a young, gifted music learner's basic psychological needs (i.e., relatedness, competence and autonomy) are not met. Another is the framework devised by Martin and colleagues (e.g., Martin and Evans, 2022) that outlines how buoyancy, resilience, and adaptability can be conceptualized as asset-oriented or strengths-based attributes that can shape a young, gifted music learners' responses to adversity, change, and uncertainty. Two other dimension relevant to talent development during childhood and adolescence would be work in positive psychology where the “broaden and build” theory of positive emotions (Fredrickson, 2001; Martin, 2004) could be adapted to expand the behavior of young, gifted musicians when they experience high levels of stress and change, in addition to conceptions related to mental wellbeing in the absence of mental illness that would allow a young musician to “flourish” (Keyes, 2007).

From the abovementioned gaps, several are considered systemic issues surrounding the music education of gifted children (see López-Íñiguez and Westerlund, in press). One of these systemic, pressing issues that was completely missing from our searches—and that explains in part why so few scholars have dealt with caring approaches to gifted education in music—is the much-needed confrontation of misconceptions that sustain narrow views that lead to the stereotyping of highly gifted children. As we mentioned at the beginning of this article, these include elitist and anti-ableists positions that refer to young, gifted music learners as if “they will survive regardless of the pressure and hardship of their training,” and conceptions that center around the outdated idea that “innate abilities do not exist and any kind of outstanding success in children can be entirely explained through their highly supportive environments” (see Brown et al., 2005; Moltzen, 2009).

### 6.2. Delimitations

The study was limited to literature published in English and Spanish, given the two authors expertise and familiarity with research paradigms in both languages. Studies published in these languages included samples of children from Europe, North America, and the Asia-Pacific. We restricted our focus to identifying caring approaches to the education of young, gifted music learners. The study results provide a framework for future efforts to devised evidence-based pedagogical models that can be used in interventions and policies aimed at catering for the needs of children gifted for music.

## 7. Conclusions

This study is among the first to critically examine existing literature concerned with caring approaches to the development and education of gifted young music learners. A scoping PRISMA review of all uncovered studies found that there is currently only a small handful of studies related to the topic of caring in music education. Of particular concern is the lack of systematic research that directly impacts on what is currently known regarding this important topic.

The article highlights the need for further research that is built on theoretical frameworks that can compare and contrast what is evident in studies of gifted young musicians with other highly demanding forms of specialization, together with a much broader approach that links this knowledge with broader national and international concerns such as human rights, ethics of care, and responsibility and laws that might protect highly gifted youth who are vulnerable due to external pressures. For instance, multidisciplinary studies are recommended to understand the complete ecosystem surrounding young, gifted music learners at micro-macro-meso levels and the role that multiple stakeholders (e.g., parents, teachers, peers, industry representatives), contexts (e.g., home, school, community, performing arenas), and variables (e.g., motivation, learning mindset, stage of development) play in it.

In conclusion, the findings of this study reaffirm the view that music education research and practice need to nurture the wellbeing of musically precocious children more adequately by expanding conceptions of talent development within an environment that takes on board (and cares for) the needs of vulnerable young music learners. This has potential to inform music education aimed at professionalism where more specialist knowledge and ethical empathic approaches to these children's education is provided (Smith, 2006; López-Íñiguez and Westerlund, in press). Such an approach follows the legal imperatives of the United Nations Convention on the Rights of the Child (brief version in UNICEF, 2010), which clearly states that gifted children in any domain deserve to have a future as healthy and agentic individuals.

## Data availability statement

The raw data supporting the conclusions of this article will be made available by the authors, without undue reservation.

## Author contributions

GL-Í conceived the original idea of the research, did the manual searches in 41 specialist research journals as well as in ARSCA and sCielo–Latindex databases, checked the searches done by a research assistant in ERIC, Scopus, Web of Science, and PsycInfo databases, and screened the eligible 506 documents in Covidence after duplicates were removed. GL-Í and GM independently screened the 67 full-text studies assessed for eligibility, formulated the conceptual ideas adapted in the manuscript, defined the search terms for the study, defined the selection/exclusion criteria for studies, and discussed about the disagreements in the final stages of screening, and provided critical feedback at all phases, discussed, and contributed to the interpretation of the results and writing of the manuscript, and approved the submitted version of the article.
